# Association of Premilitary Mental Health With Suicide Attempts During US Army Service

**DOI:** 10.1001/jamanetworkopen.2022.14771

**Published:** 2022-06-10

**Authors:** James A. Naifeh, Robert J. Ursano, Murray B. Stein, Holly B. Herberman Mash, Pablo A. Aliaga, Carol S. Fullerton, Hieu M. Dinh, Tzu-Cheg Kao, Nancy A. Sampson, Ronald C. Kessler

**Affiliations:** 1 Center for the Study of Traumatic Stress, Department of Psychiatry, Uniformed Services University of the Health Sciences, Bethesda, Maryland; 2 Henry M. Jackson Foundation for the Advancement of Military Medicine Inc, Bethesda, Maryland; 3 Department of Psychiatry, University of California San Diego, La Jolla; 4 Department of Family Medicine & Public Health, University of California San Diego, La Jolla; 5 VA San Diego Healthcare System, San Diego, California; 6 Department of Preventive Medicine and Biostatistics, Uniformed Services University of the Health Sciences, Bethesda, Maryland; 7 Department of Health Care Policy, Harvard Medical School, Boston, Massachusetts

## Abstract

**Importance:**

Approximately one-third of US soldiers who attempt suicide have not received a mental health diagnosis (MH-Dx) before their suicide attempt (SA), yet little is known about risk factors for SA in those with no MH-Dx.

**Objective:**

To examine whether premilitary mental health is associated with medically documented SA among US Army soldiers who do not receive an MH-Dx before their SA.

**Design, Setting, and Participants:**

This cohort study used data from a representative survey of soldiers in the US Army entering basic combat training from April 1, 2011, to November 30, 2012, who were followed up via administrative records for the first 48 months of service. Analyses were conducted from April 5, 2021, to January 21, 2022. Regular Army enlisted soldiers (n = 21 772) recruited from 3 US Army installations during the first week of service who agreed to have their administrative records linked to their survey responses were included.

**Exposures:**

Preenlistment lifetime history of mental disorder, suicide ideation, SA, and nonsuicidal self-injury (NSSI) as reported during the baseline survey. Service-acquired MH-Dx and sociodemographic and service-related variables were identified using administrative records.

**Main Outcomes and Measures:**

Documented SAs were identified using administrative medical records. Using a discrete-time survival framework, linear splines examined the pattern of SA risk over the first 48 months of service. Logistic regression analysis examined associations of lifetime baseline survey variables with subsequent, medically documented SA among soldiers who did vs did not receive an MH-Dx during service. Models were adjusted for time in service and sociodemographic and service-related variables.

**Results:**

Of the 21 722 respondents (86.2% male, 20.4% Black, 61.8% White non-Hispanic), 253 made an SA in the first 48 months of service (male [75.4%]; Black [22.7%], White non-Hispanic [59.9%], or other race or ethnicity [17.4%]). Risk of SA peaked toward the end of the first year of service for both those who did and did not receive an MH-Dx during service. Of the 42.3% of individuals reporting at least 1 of the 4 baseline risk factors, 50.2% received an administrative MH-Dx during service vs 41.5% of those with none, and 1.6% had a documented SA vs 1.0% of those with none. Among individuals with no MH-Dx, medically documented SAs were associated with suicide ideation (odds ratio [OR], 2.2; 95% CI, 1.1-4.4), SA (OR, 11.3; 95% CI, 4.3-29.2), and NSSI (OR, 3.0; 95% CI, 1.3-6.8). For those who received an MH-Dx, medically documented SAs were associated with mental disorder (OR, 1.4; 95% CI, 1.0-1.9), SA (OR, 3.4; 95% CI, 2.1-5.6), and NSSI (OR, 1.8; 95% CI, 1.1-2.8). Interactions indicated the only explanatory variable that differed based on history of MH-Dx was preenlistment SA (χ^2^_1_ = 4.7; *P* = .03), which had a larger OR among soldiers with no MH-Dx than among those with an MH-Dx.

**Conclusions and Relevance:**

In this study, the period of greatest SA risk and baseline risk factors for SA were similar in soldiers with and without an MH-Dx. This finding suggests that knowledge of the time course and preenlistment mental health factors can equally aid in identifying SA risk in soldiers who do and do not receive an MH-Dx.

## Key Points

**Question:**

Is premilitary mental health associated with suicide attempts among soldiers who do not receive a mental health diagnosis before their suicide attempt?

**Findings:**

This cohort study of 21 772 Regular Army enlisted soldiers found that suicide attempt risk peaked at the end of the first year of service for those who did and did not receive a mental health diagnosis. Premilitary mental health risk factors differed in the 2 groups.

**Meaning:**

The findings of this study suggest that time course and preenlistment mental health status may aid in identifying suicide attempt risk in soldiers with and without a mental health diagnosis.

## Teaser

This cohort study assesses the association between receiving a mental health diagnosis prior to enlistment and medically documented suicide attempt among current US Army soldiers who have not received a mental health diagnosis during their service.

## Introduction

Suicide rates among US Army soldiers have remained elevated since increasing sharply during the wars in Iraq and Afghanistan.^1,2,3,4^ Nonfatal suicide attempts (SAs) had a similar increase during the same period.^5^ History of mental health diagnosis (MH-Dx) is among the most common risk factors for medically documented SAs among soldiers.^6^ However, more than one-third of soldiers who attempt suicide have not previously received an MH-Dx.^7^ Identifying risk factors in those without an MH-Dx is particularly important and challenging because they are outside the military mental health care system.^7,8^

The risk factors for SA available in administrative records, such as sociodemographic and service-related characteristics, are similar for soldiers with and without a history of MH-Dx, differing primarily in the size of the odds ratios (ORs).^7^ However, little is known about the extent to which preenlistment characteristics assessed in surveys of incoming soldiers may inform our understanding of risk in these groups. Representative survey data indicate that nearly 39% of soldiers beginning basic training have a preenlistment history of a mental disorder,^9^ with preenlistment suicide ideation (SI) reported by 14.1% of individuals and SA reported by 1.9%.^10^ Although a history of mental disorder and self-injurious thoughts and behaviors is associated with an increased risk of subsequent SA,^11,12,13,14^ it is less clear whether a self-reported history of those experiences before entering military service is associated with a soldier’s SA risk, particularly in those with no history of MH-Dx during service.

In this study, we used data from the Army Study to Assess Risk and Resilience in Servicemembers (Army STARRS) New Soldier Study (NSS)^15^ to examine preenlistment risk factors for medically documented SAs among US Army enlisted soldiers during their first 48 months of service. The first 48 months of service is generally equivalent to the first term of enlistment for soldiers and is the Army career phase during which SA risk is highest.^6^ Using a representative survey of new soldiers administered during the first week of Army service, we examined preenlistment mental disorder, SI, SA, and nonsuicidal self-injury (NSSI) as risk factors for future SAs among soldiers who do and do not receive an MH-Dx during service.

## Methods

### Sample

The NSS surveyed representative samples of US Army soldiers beginning Basic Combat Training between April 1, 2011, and November 30, 2012. Soldiers were recruited within 48 hours of reporting for duty and, following written informed consent, completed a computerized self-administered questionnaire (eMethods in the Supplement). Participants did not receive financial compensation. Recruitment and consent procedures were approved by the Human Subjects Committees of all Army STARRS collaborating organizations. This report follows the Strengthening the Reporting of Observational Studies in Epidemiology (STROBE) reporting guideline for cohort studies.

The 21 772 NSS respondents considered herein represent all Regular Army enlisted soldiers who completed the self-administered questionnaire and agreed to administrative data linkage (77.1% response rate). Data were doubly-weighted to adjust for differences in survey responses among the respondents who did vs did not agree to administrative record linkage and differences in administrative data profiles between the latter subsample and the population of all new soldiers. More details on NSS weighting are reported elsewhere.^16^ Using the survey-linked administrative data, person-month records were created by coding each month of a soldier’s career for each administrative variable and allowing values to change over time for an individual soldier.^17,18^ Respondents were followed up via administrative data for up to 48 months. The actual number of administrative person-months available for NSS respondents varied because of attrition.

### Measures

#### Outcome Variable

Nonfatal SAs were identified using DoD Suicide Event Report ^19^ records and codes from *International Classification of Diseases, Ninth Revision, Clinical Modification* (*ICD-9-CM*) (codes E950-E958, indicating self-inflicted poisoning or injury with suicidal intent) and *International Statistical Classification of Diseases, Tenth Revision, Clinical Modification* (*ICD-10-CM*) (codes X71-X83, indicating intentional self-harm; codes T36-T65 and T71, with the fifth or sixth character indicating intentional self-harm; and code T14.91, indicating suicide attempt not otherwise specified)^20^ in data systems that capture health care encounter information from military and civilian treatment facilities, combat operations, and aeromedical evacuations (eTable 1 in the Supplement).

#### Explanatory Variables

Administrative personnel records (eTable 1 in the Supplement) were used to identify sociodemographic (sex, current age, race and ethnicity, educational level, and marital status) and service-related (rank, deployment status [never deployed, currently deployed, and previously deployed]) characteristics. Race and ethnicity data were included because risk for suicidal behavior varies based on sociodemographic characteristics; we combined every category other than the White non-Hispanic and Black categories into an Other category to ensure there were enough SA cases for analysis. Administrative medical records were used to create an indicator variable for MH-Dx during Army service based on *ICD-9-CM* and *ICD-10-CM* mental health diagnostic codes and mental health-related V codes and Z codes (eg, stressors/adversities and marital problems), excluding postconcussion syndrome and tobacco use disorder (eTable 2 in the Supplement). Person-months were coded such that once an MH-Dx was recorded in an individual’s records, that month and all subsequent months were coded as positive for MH-Dx.

Self-administered questionnaire responses were used to construct time-invariant baseline risk factors. Respondents self-administered a computerized version of the Composite International Diagnostic Interview screening scales^21^ and a screening version of the posttraumatic stress disorder (PTSD) Checklist ^22^ to assess 10 lifetime *Diagnostic and Statistical Manual of Mental Disorders, Fourth Edition* mental disorders: major depressive episode, bipolar I or II or subthreshold bipolar disorder, generalized anxiety disorder, panic disorder, PTSD, intermittent explosive disorder, conduct disorder, oppositional defiant disorder, substance use disorder, and attention-deficit/hyperactivity disorder. A clinical reappraisal study found good concordance between Composite International Diagnostic Interview screening scales and modified PTSD Checklist diagnoses and independent clinical diagnoses based on blinded Structured Clinical Interviews for *Diagnostic and Statistical Manual of Mental Disorders, Fourth Edition*.^23^ An indicator variable was created to identify soldiers who met criteria for any of the abovementioned disorders.

Preenlistment history of self-injurious thoughts and behaviors was assessed with a modified version of the Columbia-Suicide Severity Rating Scale,^24^ including lifetime SI (Did you ever in your life have thoughts of killing yourself? or Did you ever wish you were dead or would go to sleep and never wake up?), lifetime SA (Did you ever make a suicide attempt; that is, purposefully hurt yourself with at least some intention to die?), and lifetime NSSI (Did you ever do something to hurt yourself on purpose, but without wanting to die [eg, cutting yourself, hitting yourself, or burning yourself]?).

### Statistical Analysis

Data were analyzed from April 5, 2021, to January 21, 2022. Analyses were conducted using SAS, version 9.4 (SAS Institute Inc). Data analysis was conducted using discrete-time survival analysis with person-month the unit of analysis and a logistic link function.^17,18^ To control for changes in SA risk across time in service, we began by estimating risk (suicide attempters per 100 000 person-months) during each month of service. Splines (piecewise linear functions) were calculated based on the monthly risk estimates to identify nonlinearities in risk over the first 48 months of service. After fitting a linear function to the monthly risk estimates, we used χ^2^ difference tests, deviance, and the Akaike information criterion to assess whether knots (changes in slope) and additional linear segments improved model fit. To determine the optimal location of the first knot, we tested knots at each monthly time point beginning at month 2. We then tested for further improvements in model fit by adding knots to the intervals on either side of the first knot, repeating that process until there were no further improvements in fit. Next, a series of logistic regression models examined associations of baseline survey variables (lifetime mental disorder, SI, SA, and NSSI) with the first documented SA during the first 48 months of service. Each explanatory variable was examined separately in a model adjusting for time in service (using the spline variables), sociodemographic and service-related variables, and MH-Dx. In each multivariable model, we tested the 2-way interaction between the baseline survey variables and administrative MH-Dx. The sample was then stratified by MH-Dx and the association of each baseline survey variable with SA was examined in a separate multivariable model.

Logistic regression coefficients and confidence limits were exponentiated to obtain estimated ORs and 95% CIs. Standard errors were estimated using the Taylor series method^25^ to adjust for the weighting and clustering of the NSS data. Multivariate significance tests in the logistic regression analyses were made using Wald χ^2^ tests based on coefficient variance-covariance matrices adjusted for design effects using the Taylor series method. Statistical significance was evaluated using 2-sided design-based tests and the .05 level of significance.

## Results

### Sample Characteristics

The total cohort sociodemographic characteristics, given as weighted person-months, were as follows: male (87.6%), female (12.4%), Black (20.6%), White non-Hispanic (60.9%), other race or ethnicity (comprising American Indian/Alaska Native, Asian, Hispanic, and Native Hawaiian or other Pacific Islander individuals: 18.5%), had at least a high school education (91.0%), were not married (61.8%), were aged 21 years or older (72.9%), had E4 or higher rank (50.2%), and had never been deployed (74.0%). Sociodemographic characteristics among persons with SA in the first 48 months of service (n = 253) were as follows: male (75.4%) or female (24.6%) sex; Black (22.7%), White non-Hispanic (59.9%), or other race or ethnicity (17.4%); at least a high school education (84.4%); not married (60.8%); aged 21 years or older (61.6%); E3 or lower rank (71.0%); and never been deployed (74.5%) (Table 1).

**Table 1.** Distribution of Sample Characteristics Among a Cohort of Regular Army Enlisted Soldiers During Their First 4 Years of Service^a^

Variable	Unweighted person-months (weighted %)	
	Suicide attempt cases (n=253)	Total cohort (n=839 926)
Sex		
Female	63 (24.6)	101 936 (12.4)
Male	190 (75.4)	737 990 (87.6)
Race and ethnicity		
Black	63 (22.7)	169 884 (20.6)
White Non-Hispanic	155 (59.9)	539 990 (60.9)
Other^b^	35 (17.4)	130 052 (18.5)
Educational level		
<High school^c^	38 (15.6)	70 559 (9.0)
≥High school	215 (84.4)	769 367 (91.0)
Marital status		
Not married	152 (60.8)	526 622 (61.8)
Currently married	101 (39.3)	313 304 (38.2)
Current age, y		
≤20	93 (38.4)	218 135 (27.1)
≥21	160 (61.6)	621 791 (72.9)
Rank		
E1	30 (11.2)	50 694 (6.1)
E2	51 (17.3)	108 019 (13.0)
E3	96 (42.5)	256 985 (30.8)
≥E4	76 (29.0)	424 228 (50.2)
Deployment status		
Never deployed	197 (74.5)	625 276 (74.0)
Currently deployed	11 (5.8)	65 777 (8.0)
Previously deployed	45 (19.7)	148 873 (17.9)
Self-reported lifetime mental disorder at baseline		
Yes	123 (49.4)	295 159 (35.4)
No	130 (50.6)	544 767 (64.6)
Self-reported lifetime suicide ideation at baseline		
Yes	49 (19.9)	96 539 (11.5)
No	204 (80.1)	743 387 (88.5)
Self-reported lifetime suicide attempt at baseline		
Yes	15 (8.3)	10 148 (1.2)
No	238 (91.7)	829 778 (98.8)
Self-reported lifetime NSSI at baseline		
Yes	36 (15.9)	53 585 (6.4)
No	217 (84.1)	786 341 (93.6)
Administrative mental health diagnosis		
Yes	173 (68.3)	171 248 (20.8)
No	80 (31.7)	668 678 (79.2)

Abbreviation: NSSI, nonsuicidal self-injury.

^a^ The survey respondents considered were Regular Army enlisted soldiers (n = 21 772). Survey-linked administrative person-month records were examined through 48 months of service. The number of available person-month records for a given soldier varied because of attrition from service.

^b^ American Indian/Alaska Native, Asian, Hispanic, and Native Hawaiian or other Pacific Islander individuals. We combined every category other than the White non-Hispanic and Black categories into an Other category to ensure there were enough suicide attempt cases for analysis.

^c^ Less than high school level includes General Educational Development credential, home study diploma, occupational program certificate, correspondence school diploma, high school certificate of attendance, adult education diploma, and other nontraditional high school credentials.

Of the 21 772 unique soldiers at baseline, lifetime (preenlistment) mental disorder was reported by 37.7%; SI, 13.0%; SA, 1.7%; and NSSI, 7.3% (eTable 3 in the Supplement). The proportion of person-months (total cohort vs individuals with SA) associated with each baseline risk factor was as follows: mental disorder (35.4% vs 49.4%), SI (11.5% vs 19.9%), SA (1.2% vs 8.3%), and NSSI (6.4% vs 15.9%) (Table 1). Of the 42.3% soldiers who reported at least 1 of the 4 baseline risk factors, 50.2% received an administrative MH-Dx during service vs 41.5% of those with none of the baseline risk factors, 61.3% served for at least 48 months vs 70.7% of those with none of the baseline risk factors, and 1.6% had a documented SA during service vs 1.0% of those with none of the baseline risk factors.

### Prevalence, Risk, and Incidence of MH-Dx

**Figure 1.**
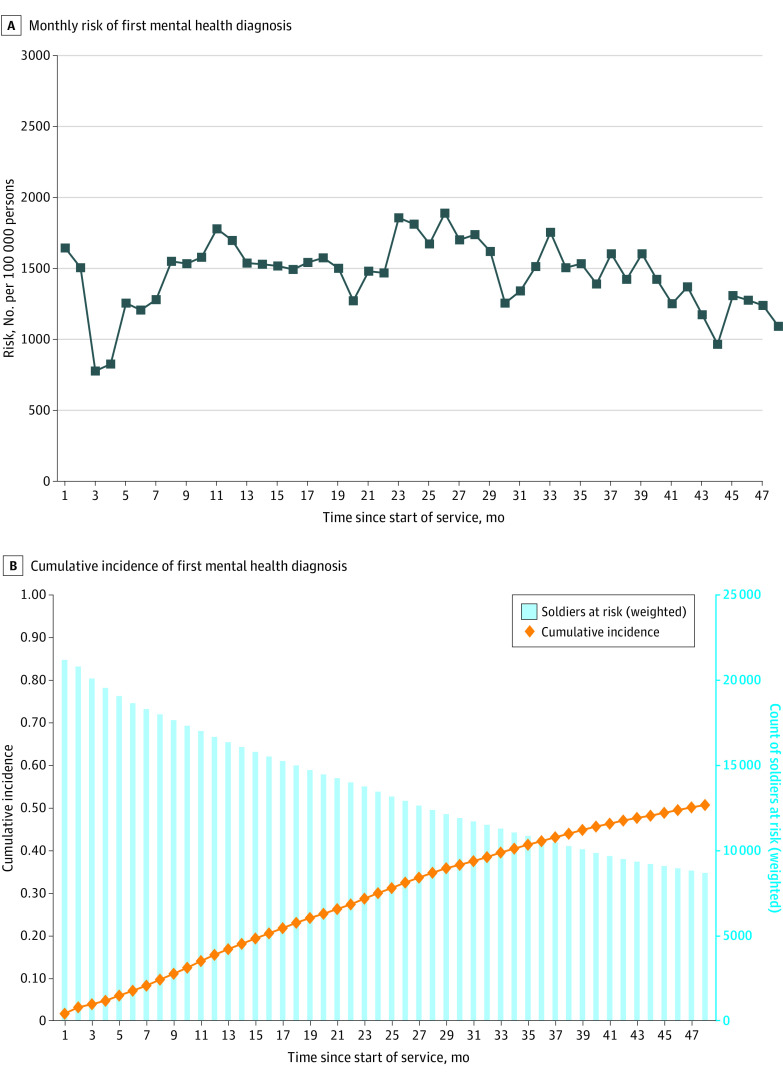
Monthly Risk and Cumulative Incidence of First Mental Health Diagnosis A, Lower rates of the first mental health diagnosis occurred near the beginning and end of the first 48 months of service. B, Cumulative incidence of diagnosis increased from 15.5% after 12 months of service to 50.7% after 48 months.

In the total cohort, a history of MH-Dx was present in 20.8% of person-month records (Table 1). The mean (SD) monthly rate of attrition from service was 842 (529) per 100 000 person-months. Approximately one-third of the cohort left service by the 48th month (eFigure in the Supplement). On average, approximately 1.5% of soldiers per month (1461 per 100 000 person-months) received a first MH-Dx, with lower rates occurring near the beginning and end of the first 48 months (Figure 1A). The cumulative incidence of first MH-Dx in the total cohort was 15.5% after 12 months of service and 50.7% after 48 months (Figure 1B). Of those who attempted suicide with no MH-Dx, 42.0% reported a preenlistment mental disorder at baseline.

### SA Risk by Time in Service

**Figure 2.**
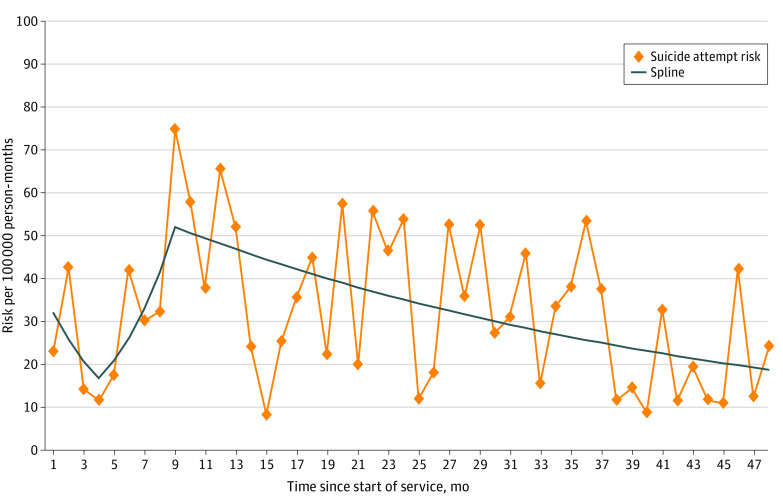
Spline Model of Suicide Attempt Risk by Time in Service Among a Cohort of Regular Army Enlisted Soldiers

We estimated SA risk in each month of service (Figure 2). Spline models identified 2 nonlinearities (at the fourth and ninth months of service). The risk of SA decreased from the start of service until the fourth month and then increased, reaching a peak in the ninth month of service. Following this peak, the risk decreased through the 48th month of service. Two-way interactions between administrative MH-Dx and each component of the spline model were nonsignificant, indicating that risk by time in service did not differ for those with and without an MH-Dx. The interaction between diagnosis and a simplified time variable that dichotomized time in service as 12 months or less vs more than 12 months was also nonsignificant.

### Baseline Survey Variables Associated With SA

In separate models that adjusted for sociodemographic and service-related variables (eTable 4 in the Supplement), medically documented SA among soldiers with no history of administrative MH-Dx was associated with self-reported lifetime SI (OR, 2.2; 95% CI, 1.1-4.4), SA (OR, 11.3; 95% CI, 4.3-29.2), and NSSI (OR, 3.0; 95% CI, 1.3-6.8) at baseline. Among those with a history of administrative MH-Dx, self-reported lifetime mental disorder (OR, 1.4; 95% CI, 1.0-1.9), SA (OR, 3.4; 95% CI, 2.1-5.6), and NSSI (OR, 1.8; 95% CI, 1.1-2.8) at baseline were associated with increased odds of a documented SA. Self-reported lifetime SA was the only baseline explanatory variable with an OR that significantly differed by MH-Dx status (χ ^2^_1_ = 4.7; *P* = .03) (Table 2). Nonstratified results are available in eTable 5 in the Supplement.

**Table 2.** Multivariable Associations of Self-reported Survey Variables With Documented Suicide Attempts Among Regular Army Enlisted Soldiers With and Without a Mental Health Diagnosis During Their First 4 Years of Service^a^

Baseline survey risk factors^c^	Previous mental health diagnosis, OR (95% CI)		Risk factor by mental health diagnosis interaction^b^	
	No	Yes	χ^2^_1_	*P* value
Lifetime mental disorder at baseline				
Yes	1.4 (0.8-2.3)	1.4 (1.0-1.9)	0.0	.88
No	1 [Reference]	1 [Reference]		
χ^2^_1_	1.5	4.5	NA	NA
*P* value	.22	.03	NA	NA
Lifetime suicide ideation at baseline				
Yes	2.2 (1.1-4.4)	1.2 (0.8-1.7)	2.6	.10
No	1 [Reference]	1 [Reference]		
χ^2^_1_	5.7	0.8	NA	NA3
*P* value	.02	.39	NA	NA
Lifetime suicide attempt at baseline				
Yes	11.3 (4.3-29.2)	3.4 (2.1-5.6)	4.7	.03
No	1 [Reference]	1 [Reference]		
χ^2^_1_	25.3	24.3	NA	NA
*P* value	<.001	<.001	NA	NA
Lifetime NSSI at baseline				
Yes	3.0 (1.3-6.8)	1.8 (1.1-2.8)	1.0	.32
No	1 [Reference]	1 [Reference]		
χ^2^_1_	7.2	5.9	NA	NA
*P* value	.008	.02	NA	NA

Abbreviations: NA, not applicable; OR, odds ratio.

^a^ The survey respondents considered were Regular Army enlisted soldiers (n = 21 772). Survey-linked administrative person-month records were examined through 48 months of service. The number of available person-month records for a given soldier varied because of attrition from service.

^b^ Each interaction was examined in a separate logistic regression model that adjusted for time in service (spline variables), sociodemographic variables (sex, race and ethnicity, education, marital status), service-related variables (rank, deployment status), and the main effects of the variable of interest and administrative mental health diagnosis.

^c^ Each explanatory variable was examined within each stratum in a separate logistic regression model that adjusted for time in service (spline variables), sociodemographic variables (sex, race and ethnicity, educational level, and marital status), and service-related variables (rank and deployment status).

Among soldiers who did not receive an MH-Dx, the model including preenlistment SA and the sociodemographic and service-related covariates had an area under the curve of 0.71, with the highest-risk ventile (5%) having a sensitivity of 18.9%, specificity of 94.8%, and positive predictive value of 45.1 per 100 000 person-months. The same model among individuals with an MH-Dx had an area under the curve of 0.70 and, within the highest-risk ventile, sensitivity of 26.7%, specificity of 94.9%, and positive predictive value of 562.2 per 100 000 person-months. Further findings are given in the eResults in the Supplement.

## Discussion

This study of medically documented SAs during the first 48 months of Army service identified similarities between soldiers with and without an MH-Dx before their SA. The pattern of risk across time in service (eg, period of greatest risk) did not differ significantly between soldiers who did or did not receive an MH-Dx. Preenlistment SA had a larger OR among soldiers without vs with an MH-Dx, whereas the ORs of the other premilitary risk factors (mental disorders, SI, and NSSI) did not differ significantly based on MH-Dx status. Our finding that lifetime mental health and self-injury risk factors reported at entry into service were similarly associated with SA among soldiers with and without an MH-Dx is an addition to research using Army administrative data that found similarities in sociodemographic and service-related risk factors for SA.^7^ This similarity suggests that preenlistment mental and behavioral health information may equally aid early intervention programs and clinical care assessments for soldiers who are and are not identified by an MH-Dx in their first 48 months of service.

We found that receiving an MH-Dx is common among enlisted soldiers; half of the individuals in this cohort received a diagnosis by the end of their fourth year of service. For soldiers with an MH-Dx, there is an opportunity for the health care system to assess suicide risk and intervene. It is more challenging to identify the roughly one-third of soldiers who made an SA while having no history of MH-Dx, which is a proportion consistent with previous research.^7^ Many of those soldiers may well have had undetected mental disorders.^26,27,28^ In this study, 42% of individuals who attempted suicide while having no history of MH-Dx reported a preenlistment mental disorder. Further work is needed to understand the extent to which those disorders continue or reemerge during service, as well as the extent to which the remaining individuals who did not receive an MH-Dx before their SA experienced a first-onset mental disorder. There are several reasons mental disorders may go undiagnosed: soldiers may not perceive a need to seek help^29^ or may not report symptoms during mental health screening,^30,31^ soldiers who screen positive may not follow up on a referral,^32^ or subsequent evaluation may not result in a diagnosis. There are opportunities for improved detection through expanded mental health screening in primary care—a universal access point to the Army health care system. Research indicates that individuals in the Army with no history of an MH-Dx who attempt suicide typically have at least 1 outpatient physical health care visit in the weeks before their attempt.^7^ Continued development of universal prevention programs is another important component of a comprehensive approach to lowering suicide risk across the population.^33^

We found SA risk varied across time in service, similarly peaking toward the end of the first year for soldiers with and without an MH-Dx. Previous analyses of records from 2004 to 2009 reported that the SA risk peaked earlier in the first year, toward the end of basic training.^6,34^ The reason for this difference is unknown; however, risk patterns might shift over time in response to changes in operational demands and in the Army’s training requirements. These findings suggest a confluence of stressors toward the end of the first year of service when many soldiers are completing training and moving to operational units. Identifying these patterns of increased risk can aid in planning preventive interventions.

A substantial proportion of soldiers entered service reporting a history of mental disorder, suicidal thoughts or behaviors, or NSSI. Our findings suggest that knowledge of these factors by the health care system may help identify increased SA risk during service, particularly among soldiers who do not receive an MH-Dx and therefore are outside the mental health care system. Given that the OR for preenlistment SA was larger among soldiers who did not vs those who did receive an MH-Dx, screening for premilitary SAs may be valuable for identifying risk in soldiers with no MH-Dx. Identifying factors that influence the persistence of mental disorders, SI, and self-injurious behaviors throughout enlistment can aid in understanding risk and resilience.^35^

Most (98.8%) soldiers who entered service with these risk factors did not attempt suicide, and nearly as many completed 48 months of service (61.3%) as those who entered with no risk factors (70.7%). Nearly half of the documented SAs were among soldiers without these preenlistment risk factors. For those entering the Army without a history of mental disorder or self-injurious thoughts and behaviors, it may be useful to identify risk factors for the first onset of such problems. Longitudinal research tracking these risk and protective factors over time, from preenlistment through postenlistment and separation from service, may improve our understanding of the course of mental health, SA risk, and resilience during military service. As part of this effort, it may be useful to identify potentially modifiable individual and unit-level factors that exacerbate or buffer the risk associated with preenlistment mental health.

We evaluated a model that considered preenlistment SA in addition to sociodemographic and service-related variables. Among soldiers with a history of an MH-Dx, the highest-risk ventile (top 5%) included 26.7% of those with an SA—a greater than 5-fold concentration of risk—with a rate of 562.2 per 100 000 person-months. Among soldiers with no history of MH-Dx, the highest-risk ventile accounted for 18.9% of those with an SA—a nearly 4-fold concentration of risk with a rate of 45.1 per 100 000 person-months. The lower SA rate in the top ventile of those who did not vs those who did receive an MH-Dx further highlights the challenge of identifying SA risk in soldiers with no MH-Dx. In addition, an individual’s risk will vary across time in service: someone who is in the top ventile during some months will likely be outside the top ventile during other months. Further study of this variation is needed to understand SA risk throughout service time.

### Limitations

This study has limitations. First, administrative data may be incomplete and/or inaccurate. Medical records are subject to errors in clinician diagnosis and coding and are unlikely to capture all SAs and mental disorders. Second, results are specific to enlisted soldiers in their first 48 months of service and to soldiers entering service during the study period. Therefore, the findings may not generalize to other service members or military eras.

## Conclusions

The findings of this study suggest the value of screening new soldiers to identify premilitary factors associated with increased SA risk during service. Previous work suggests that survey data from incoming soldiers can improve risk estimation when added to the Army’s administrative data.^36^ This study has noted that such information may be particularly useful among soldiers who do not enter the mental health care system—a segment of the population in which detection of SA risk remains an important and substantial challenge. Although the findings of the present study support screening incoming soldiers for mental disorders and self-injurious thoughts and behaviors, future research should consider dimensions of emotional, behavioral, and interpersonal functioning that are unlikely to be included in typical screening programs but may underlie a broad range of mental health problems. A key component of detection of mental health problems, particularly among soldiers with no MH-Dx, is robust mental health care within primary care.^37,38^

## Notes

Supplement. **eTable 1.** US Department of Defense and US Army Administrative Data Systems That Were Included in the Current Study **eTable 2.** *ICD-9-CM* and *ICD-10-CM* Codes Used to Identify Mental Diagnoses and Behavioral Stressors **eMethods.** Detailed Methods **eResults.** Detailed Results **eTable 3.** Baseline Distribution of Sample Characteristics in a Cohort of Regular Army Enlisted Soldiers **eFigure.** Monthly Risk and Cumulative Incidence of Attrition in a Cohort Of Regular Army Enlisted Soldiers During Their First Four Years of Service **eTable 4.** Univariable and Multivariable Associations of Sociodemographic, Service-Related, and Mental Health Diagnosis Variables With Documented Suicide Attempts Among a Cohort of Regular Army Enlisted Soldiers Over Their First Four Years of Service **eTable 5.** Univariable and Multivariable Associations of Self-report Survey Variables With Documented Suicide Attempts Among a Cohort of Regular Army Enlisted Soldiers Over Their First Four Years of Service
eReferences

## References

[1] Black SA, Gallaway MS, Bell MR, Ritchie EC. Prevalence and risk factors associated with suicides of Army soldiers 2001–2009. Mil Psychol. 2011;23(4):433-451.

[2] Gibson N, Corrigan E, Kateley K, Youmans Watkins E, Pecko JA. Surveillance of Suicidal Behavior: January Through December 2016. Division of Behavioral and Social Health Outcomes Practice, Clinical Public Health and Epidemiology Directorate, US Army Public Health Center; 2017.

[3] Pruitt LD, Smolenski DJ, Tucker J, . Department of Defense Suicide Event Report: Calendar Year 2017 Annual Report. Defense Suicide Prevention Office; DoDSER Annual Reports. 2018. Accessed December 13, 2021. https://www.dspo.mil/Prevention/Data-Surveillance/DoDSER-Annual-Reports/

[4] Tucker J, Smolenski DJ, Kennedy CH. DoDSER: Department of Defense Suicide Event Report: Calendar Year 2019 Annual Report. 2019. Accessed December 13, 2021. https://health.mil/Military-Health-Topics/Centers-of-Excellence/Psychological-Health-Center-of-Excellence/Department-of-Defense-Suicide-Event-Report

[5] Ursano RJ, Kessler RC, Heeringa SG, ; Army STARRS collaborators. Nonfatal suicidal behaviors in US Army Administrative Records, 2004-2009: results from the Army Study to Assess Risk and Resilience in Servicemembers (Army STARRS). Psychiatry. 2015;78(1):1-21. doi:10.1080/00332747.2015.1006512 26168022 PMC4503376

[6] Ursano RJ, Kessler RC, Stein MB, ; Army Study to Assess Risk and Resilience in Servicemembers Collaborators. Suicide attempts in the US Army during the wars in Afghanistan and Iraq, 2004–2009. JAMA Psychiatry. 2015;72(9):917-926. doi:10.1001/jamapsychiatry.2015.0987 26154106 PMC4558209

[7] Ursano RJ, Kessler RC, Naifeh JA, . Risk factors associated with attempted suicide among US Army soldiers without a history of mental health diagnosis. JAMA Psychiatry. 2018;75(10):1022-1032. doi:10.1001/jamapsychiatry.2018.2069 30167650 PMC6233801

[8] Britton PC, Ilgen MA, Valenstein M, Knox K, Claassen CA, Conner KR. Differences between veteran suicides with and without psychiatric symptoms. Am J Public Health. 2012;102(suppl 1):S125-S130. doi:10.2105/AJPH.2011.300415 22390586 PMC3496441

[9] Rosellini AJ, Heeringa SG, Stein MB, . Lifetime prevalence of *DSM-IV* mental disorders among new soldiers in the US Army: results from the Army Study to Assess Risk and Resilience in Servicemembers (Army STARRS). Depress Anxiety. 2015;32(1):13-24. doi:10.1002/da.22316 25338841 PMC5111824

[10] Ursano RJ, Heeringa SG, Stein MB, . Prevalence and correlates of suicidal behavior among new soldiers in the US Army: results from the Army Study to Assess Risk and Resilience in Servicemembers (Army STARRS). Depress Anxiety. 2015;32(1):3-12. doi:10.1002/da.22317 25338964 PMC5113817

[11] Borges G, Angst J, Nock MK, Ruscio AM, Kessler RC. Risk factors for the incidence and persistence of suicide-related outcomes: a 10-year follow-up study using the National Comorbidity Surveys. J Affect Disord. 2008;105(1-3):25-33. doi:10.1016/j.jad.2007.01.036 17507099 PMC2248274

[12] Joiner TE Jr, Conwell Y, Fitzpatrick KK, . Four studies on how past and current suicidality relate even when “everything but the kitchen sink” is covaried. J Abnorm Psychol. 2005;114(2):291-303. doi:10.1037/0021-843X.114.2.291 15869359

[13] Ribeiro JD, Franklin JC, Fox KR, . Self-injurious thoughts and behaviors as risk factors for future suicide ideation, attempts, and death: a meta-analysis of longitudinal studies. Psychol Med. 2016;46(2):225-236. doi:10.1017/S0033291715001804 26370729 PMC4774896

[14] Kiekens G, Hasking P, Boyes M, . The associations between non-suicidal self-injury and first onset suicidal thoughts and behaviors. J Affect Disord. 2018;239:171-179. doi:10.1016/j.jad.2018.06.033 30014957

[15] Ursano RJ, Colpe LJ, Heeringa SG, Kessler RC, Schoenbaum M, Stein MB; Army STARRS collaborators. The Army study to assess risk and resilience in servicemembers (Army STARRS). Psychiatry. 2014;77(2):107-119. doi:10.1521/psyc.2014.77.2.107 24865195 PMC4075436

[16] Kessler RC, Heeringa SG, Colpe LJ, . Response bias, weighting adjustments, and design effects in the Army Study to Assess Risk and Resilience in Servicemembers (Army STARRS). Int J Methods Psychiatr Res. 2013;22(4):288-302. doi:10.1002/mpr.1399 24318218 PMC3992816

[17] Willett JB, Singer JD. Investigating onset, cessation, relapse, and recovery: why you should, and how you can, use discrete-time survival analysis to examine event occurrence. J Consult Clin Psychol. 1993;61(6):952-965. doi:10.1037/0022-006X.61.6.952 8113496

[18] Singer JD, Willett JB. Applied Longitudinal Data Analysis: Modeling Change and Event Occurrence. Oxford University Press; 2003. doi:10.1093/acprof:oso/9780195152968.001.0001

[19] Gahm GA, Reger MA, Kinn JT, Luxton DD, Skopp NA, Bush NE. Addressing the surveillance goal in the National Strategy for Suicide Prevention: the Department of Defense Suicide Event Report. Am J Public Health. 2012;102(suppl 1):S24-S28. doi:10.2105/AJPH.2011.300574 22390595 PMC3496467

[20] Hedegaard H, Schoenbaum M, Claassen C, Crosby A, Holland K, Proescholdbell S. Issues in developing a surveillance case definition for nonfatal suicide attempt and intentional self-harm using *International Classification of Diseases, Tenth Revision, Clinical Modification* (ICD–10–CM) coded data. Natl Health Stat Report. 2018;2018(108):1-19. 29616901

[21] Kessler RC, Calabrese JR, Farley PA, . Composite international diagnostic interview screening scales for *DSM-IV* anxiety and mood disorders. Psychol Med. 2013;43(8):1625-1637. doi:10.1017/S0033291712002334 23075829

[22] Weathers FW, Litz BT, Herman DS, Huska JA, Keane TM. The PTSD Checklist: reliability, validity, and diagnostic utility. Paper presented at: Annual Meeting of the International Society for Traumatic Stress Studies; October 1993; San Antonio, Texas.

[23] Kessler RC, Santiago PN, Colpe LJ, . Clinical reappraisal of the Composite International Diagnostic Interview Screening Scales (CIDI-SC) in the Army Study to Assess Risk and Resilience in Servicemembers (Army STARRS). Int J Methods Psychiatr Res. 2013;22(4):303-321. doi:10.1002/mpr.1398 24318219 PMC4027964

[24] Posner K, Brent DA, Lucus C, . Columbia-Suicide Severity Rating Scale (C-SSRS). New York State Psychiatric Institute; 2009.

[25] Wolter KM. Introduction to Variance Estimation. Springer-Verlag; 1985.

[26] Nock MK, Dempsey CL, Aliaga PA, . Psychological autopsy study comparing suicide decedents, suicide ideators, and propensity score matched controls: results from the study to assess risk and resilience in service members (Army STARRS). Psychol Med. 2017;47(15):2663-2674. doi:10.1017/S0033291717001179 28502265 PMC5674992

[27] Nock MK, Hwang I, Sampson NA, Kessler RC. Mental disorders, comorbidity and suicidal behavior: results from the National Comorbidity Survey Replication. Mol Psychiatry. 2010;15(8):868-876. doi:10.1038/mp.2009.29 19337207 PMC2889009

[28] Cavanagh JT, Carson AJ, Sharpe M, Lawrie SM. Psychological autopsy studies of suicide: a systematic review. Psychol Med. 2003;33(3):395-405. doi:10.1017/S0033291702006943 12701661

[29] Naifeh JA, Colpe LJ, Aliaga PA, ; Army STARRS Collaborators. Barriers to initiating and continuing mental health treatment among soldiers in the Army Study to Assess Risk and Resilience in Servicemembers (Army STARRS). Mil Med. 2016;181(9):1021-1032. doi:10.7205/MILMED-D-15-00211 27612348 PMC5120390

[30] Bliese PD, Wright KM, Adler AB, Thomas JL, Hoge CW. Timing of postcombat mental health assessments. Psychol Serv. 2007;4(3):141-148. doi:10.1037/1541-1559.4.3.141

[31] Warner CH, Appenzeller GN, Parker JR, Warner CM, Hoge CW. Effectiveness of mental health screening and coordination of in-theater care prior to deployment to Iraq: a cohort study. Am J Psychiatry. 2011;168(4):378-385. doi:10.1176/appi.ajp.2010.10091303 21245086

[32] Hoge CW, Grossman SH, Auchterlonie JL, Riviere LA, Milliken CS, Wilk JE. PTSD treatment for soldiers after combat deployment: low utilization of mental health care and reasons for dropout. Psychiatr Serv. 2014;65(8):997-1004. doi:10.1176/appi.ps.201300307 24788253

[33] Wyman PA, Pisani AR, Brown CH, . Effect of the Wingman-Connect upstream suicide prevention program for Air Force personnel in training: a cluster randomized clinical trial. JAMA Netw Open. 2020;3(10):e2022532. doi:10.1001/jamanetworkopen.2020.22532 33084901 PMC7578767

[34] Ursano RJ, Kessler RC, Stein MB, ; Army STARRS Collaborators. Risk factors, methods, and timing of suicide attempts among US Army soldiers. JAMA Psychiatry. 2016;73(7):741-749. doi:10.1001/jamapsychiatry.2016.0600 27224848 PMC4937827

[35] Nock MK, Han G, Millner AJ, . Patterns and predictors of persistence of suicide ideation: results from the Army Study to Assess Risk and Resilience in Servicemembers (Army STARRS). J Abnorm Psychol. 2018;127(7):650-658. doi:10.1037/abn0000379 30335437 PMC6836677

[36] Bernecker SL, Rosellini AJ, Nock MK, . Improving risk prediction accuracy for new soldiers in the US Army by adding self-report survey data to administrative data. BMC Psychiatry. 2018;18(1):87. doi:10.1186/s12888-018-1656-4 29615005 PMC5883887

[37] Wong EC, Jaycox LH, Ayer L, . Evaluating the Implementation of the Re-Engineering Systems of Primary Care Treatment in the Military (RESPECT-Mil). RAND Corporation; 2015. 10.7249/rhq-05-02-13 PMC5158293 28083389

[38] Engel CC, Oxman T, Yamamoto C, . RESPECT-Mil: feasibility of a systems-level collaborative care approach to depression and post-traumatic stress disorder in military primary care. Mil Med. 2008;173(10):935-940. doi:10.7205/MILMED.173.10.935 19160608

