# Supplementary material for: Association of Premilitary Mental Health With Suicide Attempts During US Army Service
Source: JAMA Netw Open. 2022 Jun 10;5(6):e2214771. doi: 10.1001/jamanetworkopen.2022.14771 (PMC9187960; doi:10.1001/jamanetworkopen.2022.14771)
Supplement: Supplement. — eTable 1. US Department of Defense and US Army Administrative Data Systems That Were Included in the Current Study eTable 2. ICD-9-CM and ICD-10-CM Codes Used to Identify Mental Diagnoses and Behavioral Stressors eMethods. Detailed Methods eResults. Detailed Results eTable 3. Baseline Distribution of Sample Characteristics in a Cohort of Regular Army Enlisted Soldiers eFigure. Monthly Risk and Cumulative Incidence of Attrition in a Cohort Of Regular Army Enlisted Soldiers During Their First Four Years of Service eTable 4. Univariable and Multivariable Associations of Sociodemographic, Service-Related, and Mental Health Diagnosis Variables With Documented Suicide Attempts Among a Cohort of Regular Army Enlisted Soldiers Over Their First Four Years of Service eTable 5. Univariable and Multivariable Associations of Self-report Survey Variables With Documented Suicide Attempts Among a Cohort of Regular Army Enlisted Soldiers Over Their First Four Years of Service eReferences [file jamanetwopen-e2214771-s001.pdf]

Supplementary Online Content

Naifeh JA, Ursano RJ, Stein MB, et al. Association of premilitary mental health with suicide attempts during US Army service. *JAMA Netw Open*. 2022;5(6):e2214771. doi:10.1001/jamanetworkopen.2022.14771

eTable 1. US Department of Defense and US Army Administrative Data Systems That Were Included in the Current Study

eTable 2. *ICD-9-CM* and *ICD-10-CM* Codes Used to Identify Mental Diagnoses and Behavioral Stressors

eMethods. Detailed Methods

eResults. Detailed Results

eTable 3. Baseline Distribution of Sample Characteristics in a Cohort of Regular Army Enlisted Soldiers

eFigure. Monthly Risk and Cumulative Incidence of Attrition in a Cohort Of Regular Army Enlisted Soldiers During Their First Four Years of Service

eTable 4. Univariable and Multivariable Associations of Sociodemographic, Service-Related, and Mental Health Diagnosis Variables With Documented Suicide Attempts Among a Cohort of Regular Army Enlisted Soldiers Over Their First Four Years of Service

eTable 5. Univariable and Multivariable Associations of Self-report Survey Variables With Documented Suicide Attempts Among a Cohort of Regular Army Enlisted Soldiers Over Their First Four Years of Service

eReferences

This supplementary material has been provided by the authors to give readers additional information about their work.

© 2022 Naifeh JA et al. *JAMA Network Open.*

eTable 1. US Department of Defense and US Army administrative data systems that were included in the current study.

Database Acronym	Description
DMDC/CTS	DEFENSE MANPOWER DATA CENTER (DMDC) / CONTINGENCY TRACKING SYSTEM (CTS): Collection of activation, mobilization, and deployment data. Provides information to DoD decision makers and includes a CTS Deployment File used for tracking the location of deployed personnel.
DMDC/Master Personnel & DMDC/Transaction files	DEFENSE MANPOWER DATA CENTER (DMDC) / MASTER PERSONNEL & TRANSACTION FILES: The Active Duty Master File provides an inventory of all individuals on active duty (excluding reservists on active duty for training) at a point in time. It is a standardized and centralized database of present and past members of the active duty force. Personal data elements include social security number, education level, home of record, date of birth, marital status, number of dependents, race, ethnic group, and name. Military data elements include Service, pay grade, Armed Forces Qualification Test percentile (enlisted only), source of commission (officers only), military primary duty and secondary occupation, Unit Identification Code, months of service, duty location, Estimated Termination of Service date, basic active service date, date of current rank, pay entry base date, foreign language ability, and major command code.
DODSER	DEPARTMENT OF DEFENSE SUICIDE EVENT REPORT (DODSER): Provides risk and protective factor information for suicide events. This file contains non-fatal attempts and completed suicide cases.
MDR	MILITARY HEALTH SYSTEM DATA REPOSITORY (MDR): This database contains information about medical, dental, pharmaceutical, and ancillary claims data for both in network and purchased care as well as both inpatient and outpatient treatment. Data are collected on both Army personnel and their beneficiaries.
TMDS	THEATER MEDICAL DATA STORE (TMDS): Used to track, analyze, view and manage Soldier medical treatment information recorded in the theater of operations. Features of TMDS: accessibility and visibility of service members' deployed medical records, outpatient and inpatient treatment records created in theater facilities, treatment records from other applications, reports on movement of patients, patient status and injury/illnesses.
TRAC2ES	TRANSCOM REGULATING AND COMMAND & CONTROL EVACUATION SYSTEM (TRAC2ES): A tracking system for all medical transfers across the world for all DOD services.

© 2022 Naifeh JA et al. *JAMA Network Open.*

eTable 2. ICD-9-CM and ICD-10-CM codes[a] used to identify mental diagnoses and behavioral stressors		
**Diagnoses**	**ICD-9-CM Codes**	**ICD-10-CM Codes**
**I. Mental diagnoses**		
Adjustment disorders	309.29, 309.3, 309.4, 309.82, 309.83, 309.89, 309.9	F43.20, F43.24, F43.25, F43.29, F43.8, F43.9
Minor depression	296.82, 296.9X, 300.4, 300.5, 309.0, 309.1, 311, 313.1	F32.89, F33.8, F34.1, F34.89, F34.9. F39, F43.21
Major depression	296.2X, 296.3X	F32.0-F32.5, F32.9, F33.0-F33.4X, F33.9
Bipolar disorder	296.0X, 296.1X, 296.4X-296.7, 296.80, 296.81, 296.89, 301.13	F30.XX, F31.XX, F34.0
Anxiety state/anxiety disorder	300.0X, 300.2X, 300.3, 309.21, 309.24, 309.28, 313.0, 313.2X	F40.XXX, F41.X, F42.X, F43.22, F43.23, F93.0, F94.0
PTSD	309.81	F43.1X
ADHD/learning disorders	314.XX, 315.0X, 315.1, 315.2, 315.31, 315.32, 315.34, 315.39, 315.4, 315.5, 315.8, 315.9	F80.0-F80.4, F80.89, F80.9, F81.XX, F82, F88, F89, F90.X, H93.25, R48.0
Conduct disorder/oppositional defiant disorder	301.7, 312.4, 312.8X, 312.9, 313.81, V62.83	F60.2, F91.X, Z69.021, X69.82
Eating disorders	307.1, 307.50, 307.51, 307.59	F50.XX, F98.29
Other impulse control disorders	312.0X-312.3X	F63.XX
Alcohol-induced mental disorders/dependence/abuse	291.XX, 303.XX, 305.0X	F10.10, F10.12X, F10.14, F10.15X, F10.18X, F10.19, F10.2XX, F10.92X, F10.94-F10.99
Drug-induced mental disorders	292.XX	F11.121, F11.122, F11.14-F11.19, F11.221, F11.222, F11.24-F11.29, F11.921, F11.922, F11.94-F11.99, F12.121, F12.122, F12.15X-F12.19, F12.221, F12.222, F12.250, F12.251, F12.28X, F12.921, F12.922, F12.95X-F12.99, F13.121, F13.14-F13.19, F13.221, F13.231, F13.232, F13.24, F13.250, F13.251, F13.26-F13.29, F13.921, F13.931, F13.932, F13.94-F13.99, F14.121, F14.122, F14.14-F14.19, F14.221, F14.222, F14.23, F14.24, F14.250, F14.251, F14.28X, F14.29, F14.921, F14.922, F14.94-F14.99, F15.121, F15.122, F15.14-F15.19, F15.221, F15.222, F15.24-F15.29, F15.921, F15.922, F15.93-F15.99, F16.121, F16.122, F16.14-F16.19, F16.221, F16.24-F16.29, F16.921, F16.94-F16.99, F18.121, F18.14-F18.19, F18.221, F18.24, F18.25X-F18.29, F18.921, F18.94-F18.99, F19.121, F19.122, F19.14-F19.19, F19.221, F19.222, F19.231,

© 2022 Naifeh JA et al. *JAMA Network Open.*

		F19.232, F19.24-F19.29, F19.921, F19.922, F19.931, F19.932, F19.94-F19.99
Drug abuse without dependence	305.2X-305.9X	F11.10, F11.120, F11.129, F11.90, F11.920, F11.929, F11.93, F12.10, F12.120, F12.129, F12.90, F12.920, F12.929, F13.10, F13.120, F13.129, F13.90, F13.920, F13.929, F13.930, F13.939, F14.10, F14.120, F14.129, F14.90, F14.920, F14.929, F15.10, F15.120, F15.129, F15.90, F15.920, F15.929, F16.10, F16.120, F16.129, F16.90, F16.920, F16.929, F17.208, F17.218, F17.219, F17.228, F17.298, F17.299, F18.10, F18.120, F18.129, F18.90, F18.920, F18.929, F19.10, F19.120, F19.129, F19.90, F19.920, F19.929, F19.930, F19.939, F55.X, F11.20, F11.21, F11.220, F11.229, F11.23, F12.20, F12.21, F12.220, F12.229, F12.259, F13.20, F13.21, F13.220, F13.229, F13.230, F13.239, F13.259, F14.20, F14.21, F14.220, F14.229, F14.259, F15.20, F15.21, F15.220, F15.229, F15.23, F16.20
Drug dependence	304.XX	F16.21, F16.220, F16.229, F18.20, F18.21, F18.220, F18.229, F19.20, F19.21, F19.220, F19.229, F19.230, F19.239
Tobacco use disorder	305.1	F17.200-F17.203, F17.209, F17.210-F17.213, F17.220-F17.223, F17.229, F17.290-F17.293, Z72.0
Personality disorders	301.0, 301.10, 301.11, 301.12, 301.2X-301.9	F21, F60.0, F60.1, F60.3-F60.9, F68.10, F68.12, F69
Non-affective psychosis	295.XX, 297.X, 298.X	F20.XX, F22-F25.X, F28, F29
Somatoform/dissociative disorders	300.1X, 300.6, 300.7, 300.8X, 306.XX, 307.54, 307.8X	F44.XX, F45.XX, F48.1, F48.8, F52.5, F59, F68.11, F68.13, F68.8, G44.209
Organic mental disorders	290.XX, 293.XX, 294.0, 294.1X, 294.8, 294.9, 307.2X, 307.3, 310.0, 310.8X, 310.9, 317, 318.X, 319	F01.XX, F02.XX, F03.90, F04-F06.XX, F07.0, F07.89, F07.9, F09, F53.1, F70-F79, F95.X, F98.4, F41.83
Sexual disorders	302.XX	F52.0, F52.2X-F52.4, F52.6-F52.9, F64,1, F64,8, F64,9, F65,0-F65,4, F65,5X, F65,8X, F65,9, F66, R37, Z87.890
Sleep disorders	307.4X	F51.01-F51.03, F51.09, F51.11, F51.12, F51.19, F51.3-F51.9
Other mental disorders/mental illness	292.85, 299.XX, 300.9, 307.0, 307.52, 307.53, 307.6, 307.7, 307.9, 309.22, 310.1, 313.3, 313.82, 313.89, 313.9, 316	F48.9, F54, F84.0, F84.3, F84.9, F93.8, F93.9, F94.1-F94.9, F98.0, F98.1, F98.21, F98.3, F98.5-F98.9, F99, R45.1, R45.2, R45.5, R45.6, R45.81, R45.82
**II. Behavioral stressors**		
Suicidal ideation	V62.84	R45.851

© 2022 Naifeh JA et al. *JAMA Network Open.*

Self-damaging behavior	V69.8	N/A
Prior history of mental disorders	V11.0-V11.3, V11.8, V11.9, V66.3, V67.3	Z86.59
Indicator of impulsivity and risky behavior	V69.2, V69.3	Z72.5X, Z72.6
Stressors/adversities	V40.XX, V61.0X, V61.1X, V61.2X-V61.4X, V61.8, V61.9, V62.0-V62.5, V62.81, V62.82, V62.89, V62.9, V69.4, V69.5, V69.9	Z55.X, Z56.0-Z56.6, Z56.81, Z56.89, Z56.9, Z57.XX, Z60.0, Z60.3-Z60.9, Z62.0, Z62.1, Z62.22, Z62.29, Z62.3, Z62.6, Z62.810-Z62.812, Z62.819, Z62.82X, Z62.89X, Z62.9, Z63.XX, Z64.4, Z65.X, Z69.01X, Z69.020, Z69.1X, Z72.82X, Z72.89, Z72.9, Z73.0-Z73.81X, Z73.89, Z73.9
Traumatic stress	308.X	F43.0, R45.7

Abbreviations: ICD-9-CM, International Classification of Diseases, Ninth Revision Clinical Modification; ICD-10-CM, International Classification of Diseases, Tenth Revision Clinical Modification; PTSD, post-traumatic stress disorder; ADHD, attention deficit-hyperactivity disorder.

[a]"XX" indicates the inclusion of all fourth and/or fifth digits for that code.

© 2022 Naifeh JA et al. *JAMA Network Open.*

eMethods. Detailed Methods

Sample

The NSS survey was administered to representative samples of U.S. Army soldiers beginning Basic Combat Training (BCT) at Fort Benning, GA, Fort Jackson, SC, and Fort Leonard Wood, MO between April 2011 and November 2012. Recruitment began by selecting weekly samples of 200–300 new soldiers at each BCT installation to attend an informed consent presentation within 48 hours of reporting for duty. The presentations explained study purposes, confidentiality, and voluntary participation, then answered all attendee questions before seeking written informed consent to give a self-administered computerized questionnaire (SAQ) and neurocognitive tests and to link these data prospectively to the soldier's administrative records. These study recruitment and consent procedures were approved by the Human Subjects Committees of all Army STARRS collaborating organizations. The 21,772 NSS respondents considered here represent all Regular Army enlisted soldiers who completed the SAQ and agreed to administrative data linkage (77.1% response rate). Data were doubly-weighted to adjust for differences in survey responses among the respondents who did versus did not agree to administrative record linkage and differences in administrative data profiles between the latter subsample and the population of all new soldiers. More details on NSS weighting are reported elsewhere.[1] Using the survey-linked administrative data, person-month records were created by coding each month of a soldier's career separately for each administrative variable and allowing values to change over time for an individual soldier.[2,3] Respondents were followed via administrative data for up to 48 months. The actual number of administrative person-months available for NSS respondents varied because of attrition.

Measures

Outcome variable

SA. Non-fatal SAs were identified using administrative records from: the DoD Suicide Event Report (DoDSER),[4] a DoD-wide surveillance mechanism that aggregates information on suicidal behavior via a standardized form completed by medical providers; and codes from ICD-9-CM (E950-E958; indicating self-inflicted poisoning or injury with suicidal intent)[5] and ICD-10-CM (X71-X83, indicating intentional self-harm;

© 2022 Naifeh JA et al. *JAMA Network Open.*

and T36-T65 and T71, where the 5th or 6th character indicates intentional self-harm)[6,7] in the Military Health System Data Repository (MDR), Theater Medical Data Store (TMDS), and TRANSCOM (Transportation Command) Regulating and Command and Control Evacuating System (TRAC^{2}ES), which together provide healthcare encounter information from military and civilian treatment facilities, combat operations, and aeromedical evacuations (eTable 1).

Predictor variables

Administrative variables. Administrative personnel records (eTable 1) were used to identify time-varying socio-demographic (gender, current age, race, education, marital status) and service-related (rank, deployment status [never deployed, currently deployed, previously deployed]) characteristics. Administrative medical records were used to create an indicator variable for previous MH-Dx during Army service based on ICD-9-CM and ICD-10-CM mental health diagnostic codes and mental health-related V-codes and Z-codes (e.g., stressors/adversities, marital problems), excluding postconcussion syndrome and tobacco use disorder (eTable 2). Person-months were coded such that once a MH-Dx was recorded in an individual's records, that month and all subsequent months were coded as positive for previous MH-Dx.

Self-reported baseline survey variables. SAQ responses were used to construct time-invariant baseline predictors:

Pre-enlistment lifetime mental disorders. NSS respondents self-administered a computerized version of the Composite International Diagnostic Interview screening scales (CIDI-SC)[8] and a screening version of the PTSD Checklist (PCL)[9] to assess 10 lifetime DSM-IV mental disorders. Five internalizing disorders were assessed: major depressive episode (MDE), bipolar I-II or subthreshold bipolar disorder (BPD), generalized anxiety disorder (GAD), panic disorder (PD), and posttraumatic stress disorder (PTSD), along with five externalizing disorders: intermittent explosive disorder (IED), conduct disorder (CD), oppositional defiant disorder (ODD), substance use disorder (SUD; alcohol or drug abuse or dependence, including illicit drugs and misused prescription drugs), and attention-deficit/hyperactivity disorder (ADHD). Diagnoses were made without DSM-IV diagnostic hierarchy or organic exclusion rules. An Army STARRS clinical reappraisal study

found good concordance between CIDI-SC and modified PCL diagnoses and independent clinical diagnoses based on blinded Structured Clinical Interviews for DSM-IV (SCID).[10] An indicator variable was created to identify soldiers who met criteria for any of the above disorders.

Pre-enlistment lifetime self-injurious thoughts and behaviors (SITBs). Pre-enlistment history of SITBs was assessed with a modified version of the Columbia-Suicide Severity Rating Scale,[11] including lifetime SI ("Did you ever in your life have thoughts of killing yourself?" or "Did you ever wish you were dead or would go to sleep and never wake up?"), lifetime SA ("Did you ever make a suicide attempt; that is, purposefully hurt yourself with at least some intention to die?"), and lifetime non-suicidal self-injury ("Did you ever do something to hurt yourself on purpose, but without wanting to die [e.g., cutting yourself, hitting yourself, or burning yourself]?").

Analysis methods

Analyses were conducted using SAS version 9.4.[12] Data were analyzed using discrete-time survival analysis with person-month the unit of analysis and a logistic link function.[2,3] In order to control for changes in SA risk across time in service, we began by estimating risk (suicide attempters per 100,000 person-months) during each month of service. Splines (piecewise linear functions) were calculated based on the monthly risk estimates to identify nonlinearities in how risk changed over the course of the first 48 months of service. Specifically, after fitting a linear function to the monthly risk estimates, we used chi-square difference tests, deviance, and the Akaike Information Criterion to assess whether knots (changes in slope) and additional linear segments improved model fit. Next, a series of logistic regression models examined self-reported survey variables at baseline (lifetime mental disorder, lifetime SI, lifetime SA, lifetime NSSI) as predictors of first documented SA during the first four years of Army service. Each predictor was examined separately in a model that adjusted for time in service (using the spline variables), socio-demographic variables, service-related variables, and administrative MH-Dx. In each of those multivariable models, we tested the two-way interaction between the baseline survey predictor and administrative MH-Dx. The sample was stratified based on previous

© 2022 Naifeh JA et al. *JAMA Network Open.*

MH-Dx and each baseline survey predictor was examined separately as a multivariable predictor among person-months with and without a previous MH-Dx.

Logistic regression coefficients and their confidence limits were exponentiated to obtain estimated odds ratios (OR) and 95% confidence intervals (95% CI). Standard errors were estimated using the Taylor series method[13] to adjust for the weighting and clustering of the NSS data. Multivariate significance tests in the logistic regression analyses were made using Wald χ^2 tests based on coefficient variance–covariance matrices that were adjusted for design effects using the Taylor series method. Statistical significance was evaluated using two-sided design-based tests and the .05 level of significance.

© 2022 Naifeh JA et al. *JAMA Network Open.*

eResults. Detailed Results

Sample characteristics

In the total cohort, weighted person-months were mostly male (87.6%), White Non-Hispanic (60.9%), had at least a high school education (91.0%), not married (61.8%), at least 21-years-old (72.9%), E4 or higher rank (50.2%), and never deployed (74.0%). SA person-months (*n*=253) were mostly male (75.4%), White Non-Hispanic (59.9%), at least high school educated (84.4%), not married (60.8%), age 21 years or older (61.6%), E3 or lower rank (71.0%), and never deployed (74.5%) (Table 1).

Of the 21,772 unique soldiers at baseline, lifetime (pre-enlistment) mental disorder, SI, SA, and NSSI were reported by 37.7%, 13.0%, 1.7%, and 7.3%, respectively (eTable 3). The proportion of person-months (total cohort vs. SA cases) associated with each baseline survey predictor was: mental disorder (35.4% vs. 49.4%), SI (11.5% vs. 19.9%), SA (1.2% vs. 8.3%), and NSSI (6.4% vs. 15.9%) (Table 1). Of the 42.3% who reported at least one of the four baseline risk factors, 50.2% received an administrative MH-Dx during service (vs. 41.5% of those with none of the baseline risk factors), 61.3% served for at least 48 months (vs. 70.7% of those with none of the baseline risk factors), and 1.6% had a documented SA during service (vs. 1.0% of those with none of the baseline risk factors).

Prevalence, risk, and incidence of MH-Dx

In the total cohort, 20.8% of person-months were associated with a history of MH-Dx (Table 1). The average monthly rate of attrition from service was 842 per 100,000 person-months. Approximately one-third of the cohort left service by the 48th month (eFigure 1). On average, approximately 1.5% of soldiers per month (1,461 per 100,000 person-months) received a first MH-Dx, with lower rates occurring near the beginning and end of the first 48 months (Figure 1a). The cumulative incidence of first MH-Dx in the total cohort was 15.5% after 12 months of service and 50.7% after 48 months (Figure 1b). Of those who attempted suicide with no MH-Dx, 42.0% reported a pre-enlistment mental disorder at baseline.

© 2022 Naifeh JA et al. *JAMA Network Open.*

SA risk by time in service

We used a discrete-time survival model and linear splines to examine risk as a function of time in service (Figure 2). We first estimated risk of SA in each month of service. Spline models identified two nonlinearities (knots), one at the fourth month of service and one at the nineth month. Specifically, SA risk decreased from the start of service until the fourth month, then increased until reaching a peak in the ninth month of service. Following this peak, risk steadily decreased through the 48th month of service. Two-way interactions between administrative MH-Dx and each component of the spline model were nonsignificant, indicating that risk by time in service did not differ for those with and without a MH-Dx. It was also nonsignificant when we examined the interaction between diagnosis and a simplified time variable that dichotomized time in service as ≤ 12 months vs. > 12 months.

Administrative predictors of SA

In separate models that adjusted only for time (represented by the spline variables), significant socio-demographic predictors of SA were female gender (OR=2.3 [95% CI=1.7-3.1]), less than high school education (OR=1.8 [95% CI=1.3-2.7]), and current age of 20 or younger (OR=1.6 [95% CI=1.2-2.3]). Rank was the only significant service-related predictor, with odds of SA increasing monotonically as rank decreased from E3 (OR=3.9 [95% CI=2.4-6.5]) to E1 (OR=11.6 [95% CI=6.7-20.0]). Odds of attempt were also elevated among soldiers with a history of MH-Dx (OR=12.3 [95% CI=9.0-17.0]) (eTable 4).

Predictors were largely the same, though attenuated, when examined in multivariable models that adjusted for MH-Dx and sequentially added sociodemographic and service-related predictors. Although deployment status was nonsignificant in a model that adjusted only for time, those previously deployed had significantly elevated odds of suicide attempt in the full multivariable model (OR=1.6 [95% CI=1.1-2.3]) (eTable 4). There were no significant interactions between history of MH-Dx and the socio-demographic and service-related predictors.

© 2022 Naifeh JA et al. *JAMA Network Open.*

Baseline survey predictors of SA

In separate models that adjusted for socio-demographic and service-related variables (see eTable 4), medically documented SA among those with no history of administrative MH-Dx was associated with self-reported lifetime SI (OR=2.2 [95%CI=1.1-4.4]), SA (OR=11.3 [95%CI=4.3-29.2]), and NSSI (OR=3.0 [95%CI=1.3-6.8]) at baseline. Among those with a history of administrative MH-Dx, self-reported lifetime mental disorder (OR=1.4 [95%CI=1.0-1.9]), SA (OR=3.4 [95%CI=2.1-5.6]), and NSSI (OR=1.8 [95%CI=1.1-2.8]) at baseline were associated with elevated odds of a documented SA. Self-reported lifetime SA was the only baseline survey predictor that significantly differed by administrative MH-Dx (χ^2_1=4.7, p=0.030) (Table 2). Non-stratified results are available in eTable 5.

Among soldiers without a previous administrative MH-Dx, the model including SA and the socio-demographic and service-related covariates had an area under the curve (AUC) of 0.71, with the top ventile (5%) of predicted risk having a sensitivity of 18.9%, specificity of 94.8%, and positive predictive value (PPV) of 45.1 per 100,000 person-months. The same model among those with a previous diagnosis had an AUC of 0.70 and, within the top ventile of predicted risk, sensitivity of 26.7%, specificity of 94.9%, and PPV of 562.2 per 100,000 person-months.

© 2022 Naifeh JA et al. *JAMA Network Open.*

eTable 3. Baseline distribution of sample characteristics in a cohort of Regular Army enlisted soldiers.[a]				
	Suicide attempt cases		Total cohort	
	Unique soldiers at baseline	Weighted percentage	Unique soldiers at baseline	Weighted percentage
	N	%	N	%
Gender				
Female	63	24.6	2,959	13.8
Male	190	75.4	18,813	86.2
Race/ethnicity				
White Non-Hispanic	155	59.9	14,160	61.8
Black	63	22.7	4,364	20.4
Other	35	17.4	3,248	17.9
Education				
< High school[b]	44	18.6	2,183	10.8
High school or above	209	81.4	19,589	89.2
Marital status				
Not married	226	90.2	18,745	84.9
Currently married	27	9.8	3,027	15.1
Current Age				
≤ 20 years	164	64.9	12,793	60.0
≥ 21 years	89	35.1	8,979	40.0
Rank				
E1	108	41.5	7,992	36.7
E2	80	31.6	5,929	27.5
E3	62	25.9	6,252	28.9
E4+	3	0.9	1,599	6.8
Deployment status				
Never deployed	253	100	21,772	100
Currently deployed	0	0	0	0
Previously deployed	0	0	0	0
Self-reported lifetime mental disorder at baseline				
Yes	123	49.4	8,123	37.7
No	130	50.6	13,649	62.3
Self-reported lifetime suicide ideation at baseline				
Yes	49	19.9	2,822	13.0
No	204	80.1	18,950	87.0
Self-reported lifetime suicide attempt at baseline				
Yes	15	8.3	367	1.7
No	238	91.7	21,405	98.3
Self-reported lifetime NSSI at baseline				
Yes	36	15.9	1,588	7.3
No	217	84.1	20,184	92.7
**Total**	**253**	**100**	**21,772**	**100**

© 2022 Naifeh JA et al. *JAMA Network Open.*

[a]The survey respondents considered here were Regular Army enlisted soldiers (n = 21,772). Survey-linked administrative person-month records were examined through 48 months of service. The number of available person-month records for a given soldier varied because of attrition from service.

[b]< High School includes: General Educational Development credential (GED), home study diploma, occupational program certificate, correspondence school diploma, high school certificate of attendance, adult education diploma, and other non-traditional high school credentials.

NSSI = nonsuicidal self-injury

© 2022 Naifeh JA et al. *JAMA Network Open.*

eFigure. Monthly risk and cumulative incidence of attrition in a cohort of Regular Army enlisted soldiers during their first four years of service.

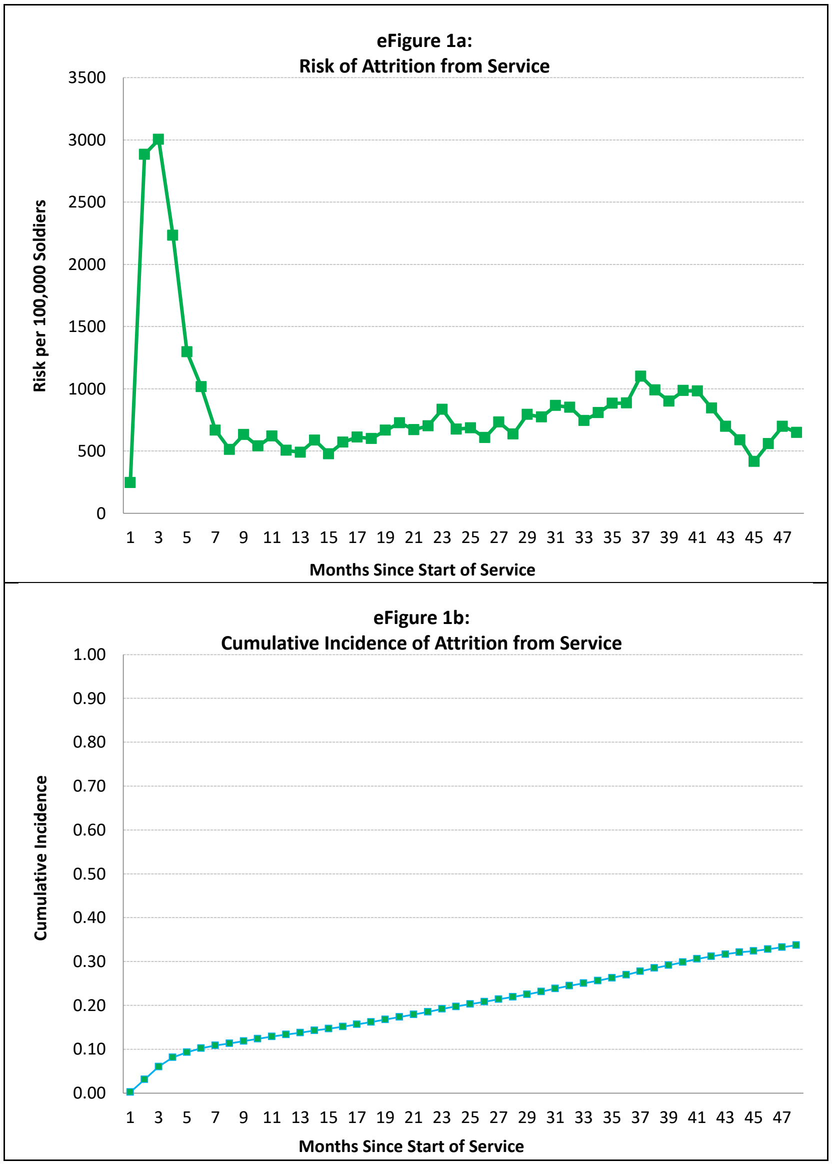

© 2022 Naifeh JA et al. *JAMA Network Open.*

eTable 4. Univariable and multivariable associations of sociodemographic, service-related, and mental health diagnosis variables with documented suicide attempts among a cohort of Regular Army enlisted soldiers over their first four years of service.[a]

	**Univariable[b]**		**Multivariable 1[c]**		**Multivariable 2[d]**	
	**OR**	**(95% CI)**	**OR**	**(95% CI)**	**OR**	**(95% CI)**
Gender						
Female	2.3*	(1.7–3.1)	1.6*	(1.2–2.2)	1.7*	(1.2–2.4)
Male	1.0	–	1.0	–	1.0	–
χ^2_1	30.3* (p<0.0001)		8.1* (p=0.005)		10.0* (p=0.002)	
Race/ethnicity						
White	1.0	–	1.0	–	1.0	–
Black	1.1	(0.8–1.6)	1.0	(0.7–1.4)	1.0	(0.7–1.4)
Other	1.0	(0.6–1.4)	0.9	(0.6–1.4)	0.9	(0.6–1.4)
χ^2_2	0.5 (p=0.77)		0.1 (p=0.96)		0.1 (p=0.95)	
Education						
< High school[b]	1.8*	(1.3–2.7)	1.8*	(1.2–2.6)	1.6*	(1.1–2.4)
High school or above	1.0	–	1.0	–	1.0	–
χ^2_1	10.4* (p=0.001)		9.2* (p=0.003)		7.0* (p=0.008)	
Marital status						
Not married	0.9	(0.7–1.2)	0.9	(0.7–1.3)	0.9	(0.7–1.3)
Currently married	1.0	–	1.0	–	1.0	–
χ^2_1	0.4 (p=0.51)		0.1 (p=0.73)		0.3 (p=0.59)	
Current age						
≤ 20 years	1.6*	(1.2–2.3)	1.7*	(1.2–2.4)	1.5*	(1.1–2.1)
≥ 21 years	1.0	–	1.0	–	1.0	–
χ^2_1	8.3* (p=0.004)		8.0* (p=0.005)		5.0* (p=0.025)	
Rank						
E1	11.6*	(6.7–20.0)			4.7*	(2.7–7.9)
E2	5.4*	(3.0–9.5)			3.1*	(1.8–5.3)
E3	3.9*	(2.4–6.5)			2.7*	(1.7–4.3)
E4+	1.0	–			1.0	–
χ^2_3	80.8* (p<0.0001)				34.0* (p<0.0001)	
Deployment status						
Never deployed	1.0	–			1.0	–
Currently deployed	0.7	(0.4–1,3)			0.9	(0.5–1.8)
Previously deployed	1.4	(1.0–2.0)			1.6*	(1.1–2.3)
χ^2_2	5.0 (p=0.08)				7.6* (p=0.023)	
Administrative mental health diagnosis						
Yes	12.3*	(9.0–17.0)	11.3*	(8.3–15.3)	10.1*	(7.4–13.8)
No	1.0	–	1.0	–	1.0	–
χ^2_1	246.6* (p<0.0001)		245.0* (p<0.0001)		211.3* (p<0.0001)	

[a]The survey respondents considered here were Regular Army enlisted soldiers (n = 21,772). Survey-linked administrative person-month records were examined through 48 months of service. The number of available person-month records for a given soldier varied because of attrition from service.

[b]Each predictor was examined separately in a logistic regression model that adjusted only for time in service (spline variables).

[c]Multivariable 1: Logistic regression model that included time in service (spline variables), socio-demographic variables (gender, race/ethnicity, education, marital status), and administratively documented mental health diagnosis.

[d]Multivariable 2: Logistic regression model that included all the variables in the Multivariable 1 model, plus service-related variables (rank, deployment status).

[b]< High School includes: General Educational Development credential (GED), home study diploma, occupational program certificate, correspondence school diploma, high school certificate of attendance, adult education diploma, and other non-traditional high school credentials.

*p < 0.05

© 2022 Naifeh JA et al. *JAMA Network Open.*

**eTable 5. Univariable and multivariable associations of self-report survey variables with documented suicide attempts among a cohort of Regular Army enlisted soldiers over their first four years of service.[a]**						
	**Univariable[b]**		**Multivariable 1[c]**		**Multivariable 2[d]**	
	**OR**	**(95% CI)**	**OR**	**(95% CI)**	**OR**	**(95% CI)**
Self-reported lifetime mental disorder at baseline						
Yes	1.8*	(1.3–2.3)	1.4*	(1.0–1.8)	1.2	(0.9–1.6)
No	1.0	–	1.0	–	1.0	–
χ^2_1	16.9* (*p*<0.0001)		5.3* (*p*=0.021)		2.2 (*p*=0.14)	
Self-reported lifetime suicide ideation at baseline						
Yes	1.9*	(1.3–2.7)	1.4	(1.0–2.1)	0.8	(0.5–1.2)
No	1.0	–	1.0	–	1.0	–
χ^2_1	6.2* (*p*=0.013)		3.6 (*p*=0.056)		1.5 (*p*=0.23)	
Self-reported lifetime suicide attempt at baseline						
Yes	7.6*	(4.6–12.5)	4.8*	(2.9–8.0)	4.3*	(2.5–7.6)
No	1.0	–	1.0	–	1.0	–
	65.4* (*p*<0.0001)		38.2* (*p*<0.0001)		26.4* (*p*<0.0001)	
Self-reported lifetime NSSI at baseline						
Yes	2.8*	(1.8–4.3)	2.0*	(1.3–3.1)	1.5	(1.0–2.5)
No	1.0	–	1.0	–	1.0	–
χ^2_1	21.5* (*p*<0.0001)		9.3* (*p*=0.003)		3.4 (*p*=0.067)	

[a]The survey respondents considered here were Regular Army enlisted soldiers (n = 21,772). Survey-linked administrative person-month records were examined through 48 months of service. The number of available person-month records for a given soldier varied because of attrition from service.

[b]Each predictor was examined separately in a logistic regression model that adjusted only for time in service (spline variables).

[c]Multivariable 1: Each predictor was examined separately in a logistic regression model that adjusted for time in service (spline variables), socio-demographic variables (gender, race/ethnicity, education, marital status), service-related variables (rank, deployment status), and administratively documented mental health diagnosis.

[d]Multivariable 2: All predictors were examined together in a logistic regression that adjusted for time in service (spline variables), socio-demographic variables (gender, race/ethnicity, education, marital status), service-related variables (rank, deployment status), and administratively documented mental health diagnosis.

*$p < 0.05$

© 2022 Naifeh JA et al. *JAMA Network Open.*

eReferences.

1. Kessler RC, Heeringa SG, Colpe LJ, et al. Response bias, weighting adjustments, and design effects in the Army Study to Assess Risk and Resilience in Servicemembers (Army STARRS). *Int. J. Methods Psychiatr. Res.* 2013;22(4):288-302. http://www.ncbi.nlm.nih.gov/pubmed/24318218

2. Willett JB, Singer JD. Investigating onset, cessation, relapse, and recovery: Why you should, and how you can, use discrete-time survival analysis to examine event occurrence. *J. Consult. Clin. Psychol.* Dec 1993;61(6):952-965. http://www.ncbi.nlm.nih.gov/pubmed/8113496.

3. Singer JD, Willett JB. *Applied longitudinal data analysis: Modeling change and event occurrence.* New York, NY: Oxford University Press; 2003.

4. Gahm GA, Reger MA, Kinn JT, Luxton DD, Skopp NA, Bush NE. Addressing the surveillance goal in the National Strategy for Suicide Prevention: The Department of Defense Suicide Event Report. *Am. J. Public Health.* 2012;102(Suppl 1):S24-S28.

5. Centers for Disease Control and Prevention. The International Classification of Diseases, ninth revision, Clinical Modification (ICD-9-CM). 2013; https://www.cdc.gov/nchs/icd/icd9cm.htm. Accessed June 10, 2021. https://www.cdc.gov/nchs/icd/icd9cm.htm

6. Centers for Disease Control and Prevention. The International Classification of Diseases, tenth revision, Clinical Modification (ICD-10-CM). 2019; https://www.cdc.gov/nchs/icd/icd10cm.htm. Accessed June 10, 2021. https://www.cdc.gov/nchs/icd/icd10cm.htm

7. Hedegaard H, Schoenbaum M, Claassen C, Crosby A, Holland K, Proescholdbell S. Issues in developing a surveillance case definition for nonfatal suicide attempt and intentional self-harm using *International Classification of Diseases, Tenth Revision, Clinical Modification* (ICD–10–CM) coded data. *National Health Statistics Reports.* February 26, 2018 2018;108.

8. Kessler RC, Calabrese JR, Farley PA, et al. Composite International Diagnostic Interview screening scales for DSM-IV anxiety and mood disorders. *Psychol. Med.* 2013;43(8):1625-1637. http://www.ncbi.nlm.nih.gov/pubmed/23075829

9. Weathers FW, Litz BT, Herman DS, Huska JA, Keane TM. The PTSD Checklist: Reliability, validity, & diagnostic utility. Paper presented at: Annual Meeting of the International Society for Traumatic Stress Studies; October, 1993; San Antonio, Texas.

10. Kessler RC, Santiago PN, Colpe LJ, et al. Clinical reappraisal of the Composite International Diagnostic Interview Screening Scales (CIDI-SC) in the Army Study to Assess Risk and Resilience in Servicemembers (Army STARRS). *Int. J. Methods Psychiatr. Res.* 2013;22(4):303-321. http://www.ncbi.nlm.nih.gov/pubmed/24318219

11. Posner K, Brent DA, Lucus C, et al. *Columbia-Suicide Severity Rating Scale (C-SSRS).* New York, NY: New York State Psychiatric Institute; 2009.

12. SAS Institute Inc. *SAS® 9.4 Software.* Cary, NC: SAS Institute Inc.; 2013.

13. Wolter KM. *Introduction to variance estimation.* New York, NY: Springer-Verlag; 1985.

© 2022 Naifeh JA et al. *JAMA Network Open.*
